# *Lactobacillus plantarum* Decreased Ammonia Emissions through Modulating Cecal Microbiotain Broilers Challenged with Ammonia

**DOI:** 10.3390/ani13172739

**Published:** 2023-08-28

**Authors:** Xiyue Liu, Guangtian Cao, Kaifan Qiu, Yingkun Dong, Caihong Hu

**Affiliations:** 1 Faculty of Science and Engineering, University of Nottingham Ningbo China, Ningbo 315100, China; hnyxl6@nottingham.ac.uk; 2 College of Standardisation, China Jiliang University, Hangzhou 310058, China; 15a1903025@cjlu.edu.cn (G.C.); aoqin1314@163.com (K.Q.); ddyykkqvq@163.com (Y.D.); 3 College of Animal Science, Zhejiang University, Hangzhou 310058, China

**Keywords:** *Lactobacillus plantarum*, ammonia emission, immune responses, antioxidant capacity, serum metabolome, ammonia challenge

## Abstract

**Simple Summary:**

Ammonia, as one pf the capital greenhouse gases, induces a negative influence on the growth performance, immune responses, and oxidative capacity of broilers. The present study indicated that *Lactobacillus plantarum* administration decreased ammonia emission, the contents of serum urea nitrogen and ammonia, fecal urease, and ammonium nitrogen. *L. plantarum* supplementation increased the concentration of major serum immunoglobulins and intestinal short-chain fatty acids, as well as the serum total-antioxidant capacity and glutathione peroxidase. Moreover, birds fed with *Lactobacillus plantarum* supplementation showed modulated cecal microflora and a changed the serum metabolome, including glyoxylate and dicarboxylate, pyruvate and thiamine metabolism, melanogenesis, and citrate cycle. From the present study, it can be seen that *L. plantarum* supplementation could be one potential method for decreasing NH_3_ emission in poultry production.

**Abstract:**

Probiotic supplementation has become a prominent method of decreasing ammonia emissions in poultry production. The present study was conducted to investigate the influence of *Lactobacillus plantarum* on ammonia emission, immune responses, antioxidant capacity, cecal microflora and short chain fatty acids, and serum metabolites in broilers challenged with ammonia. A total of 360 1-day-old yellow-feathered broilers were randomly divided into three treatment groups: birds fed with a basal diet (CON), a basal diet supplemented with ammonia (AN), and a basal diet supplemented with 2.5 × 108 CFU *L. plantarum* kg^−1^ and challenged with ammonia (LP). Data showed that *L. plantarum* supplementation decreased ammonia more than 30% from day 48, and significantly reduced the levels of serum urea nitrogen and ammonia, fecal urease, and ammonium nitrogen compared with those on CON. Compared with AN and CON treatments, LP administration increased (*p* < 0.05) the concentration of serum immunoglobulin Y (IgY), IgM, and IL-10, as well as the serum total-antioxidant capacity (T-AOC) and GSH-Px, and decreased (*p* < 0.05) IL-1β, IL-6, and TNF-α. Furthermore, birds supplemented with LP had higher (*p* < 0.05) cecal contents of short chain fatty acids (SCFAs) than AN birds and had more butyrate than CON birds. Data from 16s high throughput sequencing showed that LP supplementation significantly increased (*p* < 0.05) the Shannon and Simpson indices of bird cecal microflora, and Alloprevotella dominated the LP birds. The function prediction of cecal microflora indicated that LP treatment significantly increased alanine aspartate and glutamate metabolism, starch and sucrose metabolism, exosome, mismatch repair, homologous recombination, DNA repair and recombination proteins, and amino acid-related enzymes. The serum metabolome showed that LP supplementation significantly changed the aminoacyl-tRNA, pantothenate and acetyl-coenzyme A, arginine, phenylalanine, tyrosine and tryptophan, valine, leucine, and isoleucine biosynthesis; purine, beta-alanine, galactose, histidine, alanine, aspartate and glutamate, glyoxylate and dicarboxylate, pyruvate and thiamine metabolism, melanogenesis, and citrate cycle.

## 1. Introduction

For decades livestock production has evolved into an intensive rearing system and has induced environmental pollution. Greenhouse and odorous gases, including ammonia, methane, hydrogen sulfide, volatile organic acids, and phenolic compounds, are emitted during livestock production [1]. Non-utilized nutrients excreted through urine and feces that undergo anaerobic microbial decomposition produce a high content of odorous compounds, which leads to poor performance of broilers and various diseases. During the host metabolism, only 30–50% of the consumed nitrogen is used and excreted as urinary ammonia, which mainly becomes the potential source of ammonia emission. Particularly, a high indoor level of ammonia induced a decrease in growth performance, poor immune responses, increased organ damage, and a high mortality risk in broilers [2]. It was confirmed that under either aerobic or anaerobic conditions, ammonia is produced by the microbial decomposition of N-based compounds [3]. Much of the ammonia released from manure comes from the hydrolysis of urea [4] or the breakdown of uric acid [5]. Furthermore, ammonia can disturb the balance of the intestinal microflora and aggravate inflammatory responses in livestock [6,7].

Among the prominent methods, including physical, chemical, and biological removal of ammonia, microbial deodorization technologies are the most widely used [8]. Probiotics were shown to enhance nutrient digestion, modulate intestinal microflora [9], and reduce ammonia emissions in broilers [4,10]. Studies indicate that probiotics provide material and technical support for the healthy development of the poultry industry and odor control, while effectively inhibiting malodor by reducing gut pH and enhancing nitrogen application [11]. Kim et al. (2006) screened one strain of *L. plantarum*, which could effectively remove ammonia reaching 98.5% in poultry production [12,13]. Meng et al. (2019) reported that *L. plantarum* administration can lead to significantly less fecal ammonia [14]. Supplementation with *L. plantarum* also increases the plasma levels of IgA and IL-10 and reduces IL-1β, thereby enhancing the health status of broilers [15]. However, studies on the influence of LP treatment on ammonia metabolism, serum metabolome, and changes in the specific cecal microbiota of broilers are limited, while our previous study also confirmed that dietary Bacillus amyloliquefaciens changed the caecal metabolome, including the amino acid and glyceride metabolism in broilers [16]. Serum metabolomics also has been proven as a new method to predict broilers’ digestive efficiency [17]. Hence, the present study was conducted to investigate the effects of LP on ammonia emission, immune responses, antioxidant capacity, serum metabolomics, and cecal microflora in broilers exposed to ammonia.

## 2. Materials and Method

### 2.1. Experimental Design

The following experimental procedures were conducted according to the guidelines of the Animal Management Rules of the Ministry of Health of the People’s Republic of China and were approved by the Animal Management Committee of the Institute of Animal Sciences, Zhejiang University (Hangzhou, China). A total of 480 one-day-old, yellow-feathered broilers were randomly divided into four treatment groups with eight replicates of 15 birds per pen. Birds were fed a basal diet (CON), a basal diet supplemented with 2.5 × 10^8^ cfu microencapsulated *L. plantarum* (ZJLP-016) kg^−1^ (Lp), a basal diet and exposed with ammonia on d 56 (AN), and a basal diet supplemented with 2.5 × 10^8^ cfu microencapsulated *L. plantarum* (ZJLP-016) kg^−1^ and exposed with ammonia on d 56 (LP). From day 56, AN and LP broilers were exposed to nitrogen cubes (10.0 m × 5.0 m × 4.0 m, length × width × height) for 1 h for 7 days and the ammonia concentration was maintained at 35 mg/m^3^, in which the explosion location was the same house for CON and LP broilers. All birds raised in cages were provided free access to food and water for a total of 63 days. Initially, the room temperature was set at 35 °C and then gradually decreased to 27 °C by 2 °C reduction/week according to NRC 2004. The basal diet composition and nutritional content are shown in Table 1. At the end of the study, one broiler per replicate of CON, AN, and LP was randomly selected and euthanized, and blood was obtained from the inferior pterygoid vein. The fresh manure and cecal digesta were collected and stored in sterile cryopreservation tubes at −80 °C for further measurement.

### 2.2. Determination of Ammonia

The ammonia content was detected in broiler houses following the method of Harper et al. (2010) [18], who measured three replicates of the upper, middle, and ground atmospheres of poultry house using open-path line-averaging laser spectrometry (Boreal Laser Inc., Spruce Grove, AB, Canada). The measurements were conducted at 8:00 am, and the averages of the upper, middle, and group atmospheres were considered as the daily data.

### 2.3. Determination of Serum Indexes

The blood was collected from carotid artery and centrifuged for 10 min (3000× *g*, 25 °C), and the obtained serum was stored at −20 °C. The levels of serum immune parameters, including IgA, IgY, IgM, IL-1β, IL-6, IL-10, and TNF-α, and the oxidation resistance indices, including total antioxidant capacity (T-AOC), malonyldialdehyde (MDA), superoxide dismutase (SOD), and glutathione peroxide (GSH-Px), were determined using specific commercial test kits (Aoqing Biotech Co., Ltd., Nanjing, China) according to the manufacturer’s instructions.

### 2.4. Determination of Ammonia Metabolism Related Parameters

The levels of fecal urea nitrogen, uric acid, urease, ammonium nitrogen, serum ammonia, uric acid, urea nitrogen, and xanthine oxidase (XOD) were measured using commercial kits (Nanjing Jiancheng, Nanjing, China) following the manufacturer’s instructions. The fresh feces were collected from each replicate and stored at −20 °C until further detection. Combining 1 g fecal sample with 4 mL dd H_2_O, the mixture was completely mixed and then heated in a water bath at 50 °C for 30 min. Subsequently, the samples were shaken for 30 min (40 °C, 180 r/min), from which the supernatant was collected after centrifugation (3000× *g*, 5 min) for the next measurement.

### 2.5. Cecal SCFAs Content

The cecal SCFAs content was examined using gas chromatography (GC). Briefly, the external standards of major SCFAs were purchased from Sigma-Aldrich (Shanghai, China), which consisted of acetic acid, butyrate, propionic acid, isovalerate, isobutyric acid, and valerate. The mixture together with 0.5 g cecal digesta and 25% phosphorous acid (*m*/*v*, 1:5) was completely vibrated and centrifuged. Subsequently, the supernatant was injected into the GC network (GC 7890A, Agilent Technologies, Santa Clara, CA, USA), equipped with an HP-FFAP column (30 m × 0.53 mm × 1.00 um, Agilent Technologies) and flame ionization detector.

### 2.6. Cecal Microbial 16S Sequencing

A total of 24 cecal content samples were selected for 16s high throughput sequencing. 16S sequencing was performed on the Illumina NovaSeq platform at Novogene Bioinformatics Technology Co., Ltd. (Beijing, China), where the paired ending was performed after building the fragment genomic library. The V3–V4 variable region of 16S rRNA genes was amplified for the analysis of cecal microflora structure using specific primers (5′-ACTCCTACGGGAGAGGCAGCA-3′ and 5′-GGACTACHVGGGTWTCTAAT-3′). The data analysis was similar to that described by Zhu et al. (2018) [17]. Briefly, after the clustering and analysis of clean data, a Venn graph was made to figure out the common and unique Operational Taxonomic Units (OTUs) generated from clean reads using Vsearch software (version 2.4.2) with 97% similarity, and the alpha and beta diversity were used to analyze the between-group and within-group differences. The ternary plot analyzed the divergence of microfloral relative abundance of all treatment birds at the genus and species levels, and the top 12 strains were selected. Furthermore, the Metastat analysis was selected to analyze the relative abundance difference of different strains between all treatment groups at the genus level. Phylogenetic investigation of communities by reconstruction of unobserved states (PICRUSt) was used to predict the microfloral biological function between different groups.

### 2.7. Serum Metabolome

The nontargeted serum metabolome was analyzed by Luming Biotechnology Co., Ltd. (Shanghai, China). The serum samples combined with *L*-2-chlorophenylalanine were vortexed and added to a mixture of methanol and acetonitrile (2/1, vol/vol). After extraction and centrifugation, centrifugal freeze concentration was used to dry the supernatant. Subsequently, the resultant mixture with added methoxylamine hydrochloride was completely vortexed for 2 min and incubated for 90 min at 37 °C. Finally, the derivatized samples were detected on an Agilent 7890B GC system (Agilent Technologies Inc., Santa Clara, CA, USA) equipped with an HP-5MS fused-silica capillary column (30 m × 0.25 mm × 0.25 μm, Agilent J and W Scientific, Folsom, CA, USA) and an Agilent 5977 B MSD system.

The raw data obtained were detected, identified, aligned, and filtered to derive the data matrix. Thereafter, principal component analysis (PCA) of the data matrix was performed using R software (Version 2.15.3), and orthogonal Partial Least-Squares-Discriminant Analysis (OPLS-DA) was conducted to screen the distinguished metabolites within all groups. Then, the two-tailed Student’s t-test was used to identify the differential metabolites within all groups, with VIP values > 1.0 and *p* < 0.05. The Kyoto Encyclopedia of Genes and Genomes (KEGG) database (http://www.kegg.jp, accessed on 12 May 2023), SIMCA-P (version 13.0, Sartorius Stedim Biotech Ltd., Umea, Sweden), and MultiExperiment Viewer (version 4.8, Quantitative Biomedical Research Center, Boston, MA, USA) were used to assess the significantly altered metabolites (*p* < 0.05) and metabolic routes.

### 2.8. Statistical Analysis

Significant differences within all data were analyzed using one-way ANOVA with Tukey’s test, which was performed using IBM SPSS Statistics (Version 25.0, IBM Corp., Armonk, NY, USA). The *p* value < 0.05 was considered as statistically significant; 0.05 < *p* < 0.1 was considered as a tendency, but not a significant difference. The metabolomic data used in the analysis for distinguishing pathways were those that exhibited a fold change >2 and had a *p*-value < 0.05, as determined by a *t*-test. Graphs were generated using GraphPad Prism 5.0 (GraphPad Software, Inc., San Diego, CA, USA).

## 3. Results

### 3.1. Ammonia Concentration

**Figure 1:**
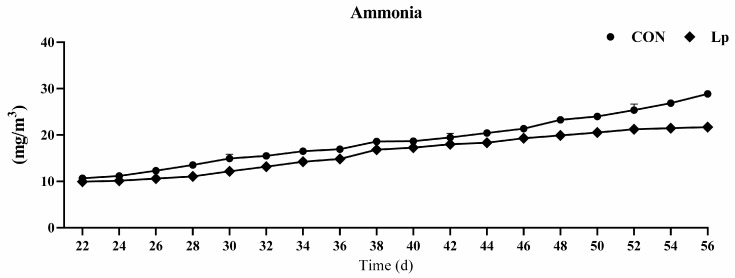
Effects of *Lactobacillus plantarum* on ammonia concentration in broilers house. CON represents birds fed with basal diet, LP represents birds fed with basal diet added *L. plantarum*. *n* = 8.

The indoor ammonia concentration was measured every two days, as shown in Figure 1. From days 22 to 46, no obvious difference was found between the two treatment groups, except from days 28 to 32. From day 48, supplementation with LP significantly decreased ammonia concentration compared with that of the CON treatment. From day 48, the LP supplementation decreased more than 30% ammonia compared with CON supplementation.

Studies have found that probiotics are effective for inhibiting growth of pathogenic microorganisms, reducing the emission of ammonia, and transforming odorous gas [19,20,21], and thereby promoting the growth performance of livestock [9,19]. Probiotics can reduce the intestinal urease-generating microbiota by generating antimicrobial barnase and mersacidin [22,23], thereby attenuating the release of ammonia. The administration of compound probiotics comprising of *L. plantarum*, *Pichia guilliermondii*, *B. coagulans*, and *B. subtilis* was confirmed to decrease the ammonia emissions by more than 39% in egg-laying hens [11]. Meng et al. (2019) found that *L. plantarum* treatment significantly decreased the ammonia concentration and fecal pH in cyclophosphamide-treated mice [14]. Similarly, the present study confirmed that the LP administration significantly decreased the ammonia emission, which especially decreased more than 30% after d 21, although the combination of the three *Lactobacillus* strains administration at 1.0 × 10^12^ CFU/kg feed did not significantly change the fecal ammonia, ammonium-nitrogen, or total *n* content of egg-laying hens, nor the serum ammonia and uric acid [24]. Different strains and dosages of *Lactobacillus* have different roles in decreasing ammonia outcomes in poultry production.

### 3.2. Ammonia Metabolism Related Parameters

**Figure 2:**
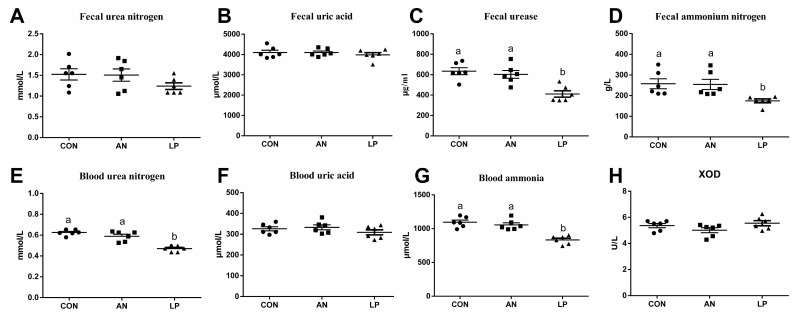
Effects of *Lactobacillus plantarum* on ammonia metabolism related parameters content of broilers exposed to ammonia. (**A**) fecal urea nitrogen, (**B**) fecal uric acid, (**C**) fecal urease, (**D**) fecal ammonia nitrogen, (**E**) blood urea nitrogen, (**F**) blood uric acid, (**G**) blood ammonia, (**H**) XOD. CON represents birds fed with basal diet, AN represents birds fed with basal diet and exposed to ammonia, LP represents birds fed with basal diet added *L. plantarum* and exposed into ammonia. a, and b represent significant differences between groups. Broilers were regarded as experimental units, each treatment with *n* = 8. ● represents CON, ■ represents AN, ▲ represents LP.

The effects of LP supplementation on fecal and serum amino-metabolism-related parameters are shown in Figure 2. Compared with those of the CON and AN treatment groups, LP birds had significantly lower (*p* < 0.05) fecal urease and ammonium nitrogen concentrations. Furthermore, LP supplementation dramatically decreased (*p* < 0.001) the blood urea nitrogen and ammonia content compared to those in CON and AN treatments. No significant differences were found in the concentrations of urea nitrogen, uric acid in feces, uric acid, or XOD in the blood.

It is well known that urea nitrogen, uric acid, and protein obtained from diets rich in amino acids make up the ammonia in poultry houses [2]. The largest quantity of nitrogen in poultry urine is comprised of uric acid and ammonia [25]. Furthermore, uric acid represents 50–60% of the total *n* content of poultry excreta, in view of the available research data [26]. Uric acid degradation can minimize ammonia volatilization and improve ammonia retention in broiler production [2]. Poultry breeds with higher growth rates and egg production have reduced ammonia retention and excretion [27]. Our data showed that LP supplementation dramatically decreased the levels of urea nitrogen and ammonia in blood, fecal urease, and ammonium nitrogen. According to Patrick and Glasswell (2015) [28], a large amount of uric acid in manure is transferred to ammonia through a series of reactions catalyzed by uricase and urease enzymes. Inhibitors of enzymes promote urea aggregation and block the growth of intestinal microbiota and enzyme activity, thereby reducing ammonia volatilization [29].

### 3.3. Serum Immune Responses

**Figure 3:**
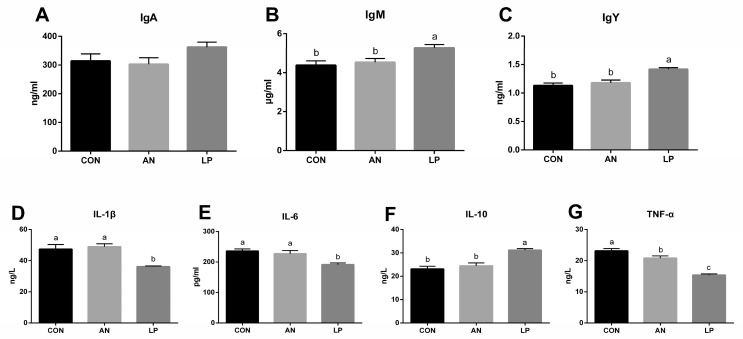
Effects of *Lactobacillus plantarum* on serum immune responses in broilers exposed to ammonia. (**A**) IgA, (**B**) IgM, (**C**) IgY, (**D**) IL-1β, (**E**) IL-6, (**F**) IL-10, (**G**) TNF-α. CON represents birds fed with basal diet, AN represents birds fed with basal diet and exposed to ammonia, LP represents birds fed with basal diet added *L. plantarum* and exposed into ammonia. a, b, and c represent significant differences between groups. *n* = 8.

The effects of LP supplementation on serum immune responses are shown in Figure 3. The LP supplementation significantly increased (*p* < 0.05) the content of IgY and IgM compared to those in CON and AN treatments. No significant difference in IgA was found among the treatment groups. Compared with those in AN and CON treatments, LP supplementation significantly decreased (*p* < 0.05) the serum levels of IL-1β, IL-6, and TNF-α, but significantly increased (*p* < 0.05) IL-10.

Immunoglobulins, consisting of IgA, IgM, and IgY, play a crucial role in immune responses and mucosal defense in broilers [15]. Ammonia reduces immune function by altering inflammatory indices [30]. Wu et al. (2019) found that probiotic *L. plantarum* supplementation improved the hepatic and jejunal mucosal expression of IL-10 and jejunal sIgA content in broilers [31]. The postbiotics derived from *L. plantarum* treatment were confirmed to increase the plasma IgM content of broilers, although they did not influence IgA levels [32]. Dietary LP supplementation was also found to reduce the mRNA expression of the ileal mucosal TNF-α gene [33]. Consistent with the above studies, the present study indicated that adding LP induced a decrease in serum IL-1β, IL-6, and TNF-α, and increased IgM, IgY, and IL-10 concentrations, suggesting that dietary LP was effective for stimulating the humoral immune response in broilers challenged with ammonia.

### 3.4. Serum Antioxidant Capacity

**Figure 4:**
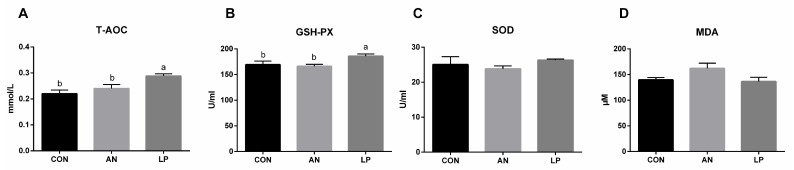
Effects of *Lactobacillus plantarum* on serum antioxidant capacity of broilers exposed to ammonia. (**A**) T-AOC, (**B**) GSH-Px, (**C**) SOD, (**D**) MDA. CON represents birds fed with basal diet, AN represents birds fed with basal diet and exposed to ammonia, LP represents birds fed with basal diet added *L. plantarum* and exposed into ammonia. a, and b represent significant differences between groups. *n* = 8.

The effects of LP supplementation on serum antioxidant capacity are shown in Figure 4. Compared with those in CON and AN treatment groups, LP supplementation significantly increased (*p* < 0.05) the content of T-AOC and GSH-Px in broilers. No significant differences were found in the SOD and MDA levels among the treatment groups.

The major antioxidases, as the first defence barrier, including SOD, CAT, and GSH-Px, resist oxidative attack [34]. T-AOC is a comprehensive parameter for assessing antioxidant enzyme concentrations in animals [35], whereas MDA levels directly reflect the degree of lipid oxidative impairment. Probiotics can enhance the oxidation resistance of the hosts by removing redundant free radicals, thus resulting in higher GSH-Px and CAT activities and lower SOD activity [36]. Xu et al. (2020) found that LP administration significantly decreased the total serum SOD levels of egg-laying chickens challenged with *Clostridium perfringens*. Similar to that in other studies, LP supplementation induced a significantly higher serum content of T-AOC and GSH-Px in broilers challenged with ammonia in the present study [33]. In fact, gaseous ammonia enters the blood and increases its concentration of blood ammonia, thereby mediating oxidative stress responses by inducing redox homeostasis [37]. Gao et al. (2013) also confirmed that *L. plantarum* activated the signal pathway of Nrf2, and thereby played an antioxidative role [38]. Based on these data, LP administration enhanced the antioxidant capacity of broilers challenged with ammonia by enhancing their major antioxidases.

### 3.5. Fecal Short Chain Fatty Acids

**Figure 5:**
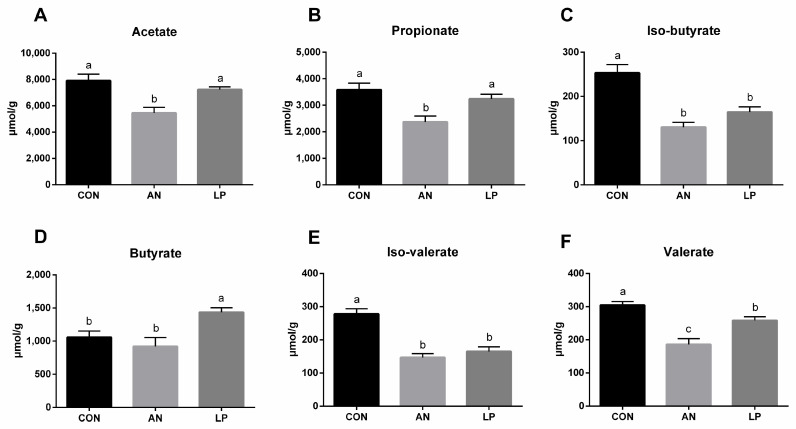
Effects of *Lactobacillus plantarum* on fecal SCFAs content of broilers exposed to ammonia. (**A**) acetate, (**B**) propionate, (**C**) iso-butryrate, (**D**) butyrate, (**E**) iso-valerate, (**F**) valerate. CON represents birds fed with basal diet, AN represents birds fed with basal diet and exposed to ammonia, LP represents birds fed with basal diet added *L. plantarum* and exposed into ammonia. a, b, and c represent significant differences between groups. *n* = 8.

Ammonia challenge significantly decreased the fecal content of SCFAs compared to that in CON birds (Figure 5). All birds supplemented with LP had a higher content of SCFAs compared with ammonia challenged birds. The LP supplementation significantly increased (*p* < 0.05) the fecal level of butyrate compared to that in CON birds, while it decreased (*p* < 0.05) valerate, iso-valerate, and iso-butyricum.

As a particular type of microfloral metabolite fermented by dietary fibers, SCFAs produced by the microbiota were shown to influence animal physiology [39]. Supplementation with *L. plantarum* increased fecal SCFA levels in broilers [40]. The *L. plantarum* treatment significantly increased total cecal SCFAs levels in broilers challenged with DON. Our data showed that LP administration significantly increased the cecal concentration of butyrate while decreasing valerate, iso-valerate, and iso-butyricum. Compared to cyclophosphamide-treated mice, *L. plantarum* treatment significantly induced the production of acetic, propionic, butyric, and valeric acids [14]. Additionally, Bacteroides, the major beneficial microorganisms, can ferment carbohydrates to induce more SCFAs, including acetic, propionic, isobutyric, and isovaleric acids [41,42]. The present 16S sequencing data showed that supplementation with LP significantly increased the relative abundance of the *Bacteroides* genus in the cecum of broilers, which also supported these findings. Furthermore, a higher concentration of SCFAs increased intestinal acidity, which boosted the application of ammonia by intestinal microflora using it as a nitrogen source, thereby downgrading the ammonia level [43]. Conclusively, LP administration reduced ammonia emission by modulating cecal microflora and cecal SCFAs, and therefore serum metabolites enhanced the antioxidant capacity and immune responses of broilers challenged with ammonia.

### 3.6. Cecal Microflora

**Figure 6:**
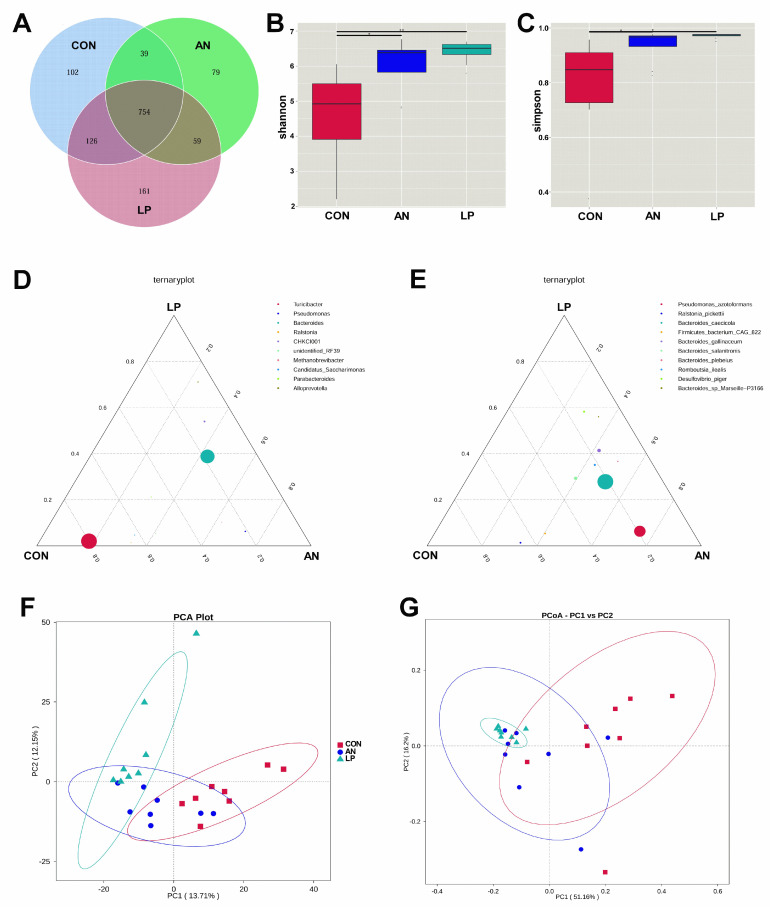
Effects of *Lactobacillus plantarum* supplementation on cecal microflora in broilers exposed to ammonia. (**A**) Venn graph, (**B**) shannon index, (**C**) simpson index, (**D**) ternaryplot on genus level, (**E**) ternaryplot on specie level, (**F**) PCA analysis, (**G**) PCoA analysis. CON represents birds fed with basal diet, AN represents birds fed with basal diet and exposed to ammonia, LP represents birds fed with basal diet added *L. plantarum* and exposed into ammonia. “*” represents *p* value < 0.05, “**” represents *p* value < 0.01. *n* = 8.

All birds shared 754 OTUs from the Venn dram in all treatments (Figure 6A). LP supplementation significantly increased (*p* < 0.05) the Shannon and Simpson indices compared with those in the CON and AN treatments (Figure 6B,C). From the ternary plot at the genus level, *Turicibacter*, *Candidatus Saccharimonus*, and *Ralstonia* dominated the cecal microflora of the CON birds, Pseudomonas dominated the AN birds, and *Alloprevotella* dominated the LP birds. Furthermore, at the species level, *Ralstonia Pickettii* dominated the cecal microflora of the CON broilers, *Desulfovibrio piger*, *Bacteroides gallinaceum*, *Bacteroides plebeius*, and *Romboutsia ilealis* dominated AN and LP birds, and *Pseudomnus azotoformans* dominated the AN birds. Additionally, cecal samples of LP birds were well separated from those of the CON and AN birds, whereas the main areas of CON and AN birds were also separated from each other from the PCA and PCoA plots.

**Figure 7:**
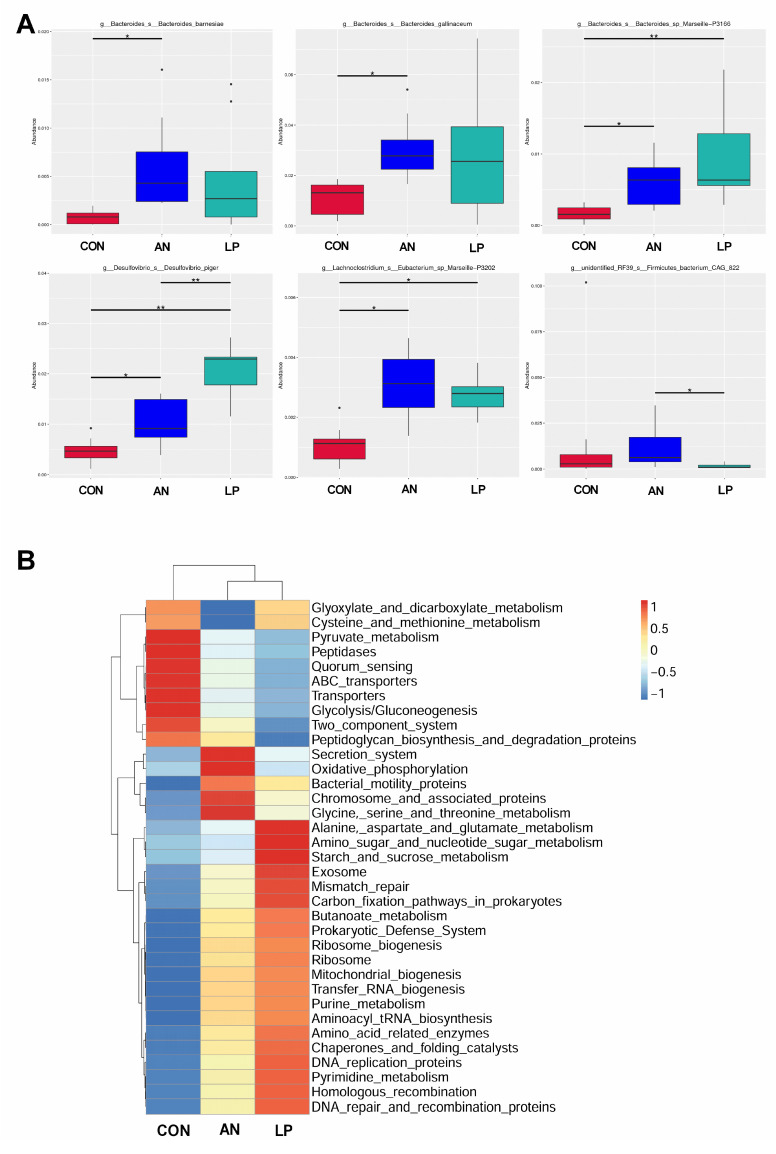
Effects of *Lactobacillus plantarum* supplementation on cecal microflora in broilers exposed to ammonia. (**A**) *t*-test on genus level, (**B**) PICRUSt analysis. CON represents birds fed with basal diet, AN represents birds fed with basal diet and exposed to ammonia, LP represents birds fed with basal diet added *L. plantarum* and exposed into ammonia. “*” represents *p* value < 0.05, “** ” represents *p* value < 0.01. *n* = 8.

The *t*-test showed that the relative abundance of *Bacteroidesbarnesiae* and *Bacteroidesgallinaceum* in AN birds was significantly higher (*p* < 0.05) than that in CON birds (Figure 7A). After supplementation with LP, the relative abundances of *Lachnoclostridium Eubacterium*, *Bacteroides* sp. *Marseille*, and *Desulfovibrio_s_Desulfovibrio_piger* were significantly higher (*p* < 0.05) than those of the CON and AN birds. Furthermore, LP birds had a significantly higher (*p* < 0.05) *Desulfovibrio_s_Desulfovibrio_piger* concentration than AN birds. *Firmicutes_bacterium*_CAG in the LP group was significant higher (*p* < 0.05) than in the AN group. A heatmap of the function prediction of cecal microflora indicated that scores of alanine asparate and glutamate metabolism, starch and sucrose metabolism, exosome, mismatch repair, homologous recombination, DNA repair and recombination proteins, carbon fixation pathways in prokaryotes, and amino acid-related enzymes in the LP treatment groups were higher than those in the CON and AN birds (Figure 7B). Oxidative phosphorylation, chromosome and associated proteins, and secretion systems in the AN birds were higher than those in the CON and LP birds.

The intestinal tract is an important digestive organ, in which the microorganism play key roles in absorbing nutrients and preventing disease [6]. According to previous studies, the reduction in NH_3_ emissions may have occurred due to increased utilization of nutrients and alteration of the intestinal microbiota in broilers [44]. Probiotic supplementation modulates the excreta fecal microfloral composition, which results in the lowering of excreta ammonia emission and the improvement of ammonia digestibility [45]. Moreover, Gates (2000) confirmed that the undigested protein is converted to ammoniacal nitrogen through bacterial activity, resulting in ammonia and ammonium (NH_4_^+^) formation in poultry [46]. The present data showed that either LP administration or ammonia treatment dramatically changed the cecal microflora structure through PCA and PCoA analysis. Additionally, the LP supplemented broilers significantly enhanced the alpha diversity, including the Shannon and Simpson index, and increased the relative abundance of *Lachnoclostridium*, and *Desulfovibrio piger* compared with CON treatment. Similarly, a study conducted by Inna Burakova et al. (2022) confirmed that the mixture of *L. acidophilus*, *L. plantarum,* and *L. fermentum* raised the relative abundance of *Lachnoclostridium*, which is beneficial for intestinal health [47]. Kalina Duszka (2022) has confirmed that *Desulfovibrio piger* is a sulfate-reducing bacterium [48]. From the KEGG data, the LP treatment significantly improved the function prediction score of alanine asparate and glutamate metabolism, starch and sucrose metabolism, exosome, mismatch repair, homologous recombination, DNA repair and recombination proteins, carbonfixation pathways in prokaryotes, and amino acids related enzymes than other treatments. The PICRUSt showed that ammonia challenge also increased the oxidative phosphorylation, chromosome and associated proteins, and secretion system score more than other treatments, based on KEGG data base.

### 3.7. Serum Metabolome

**Figure 8:**
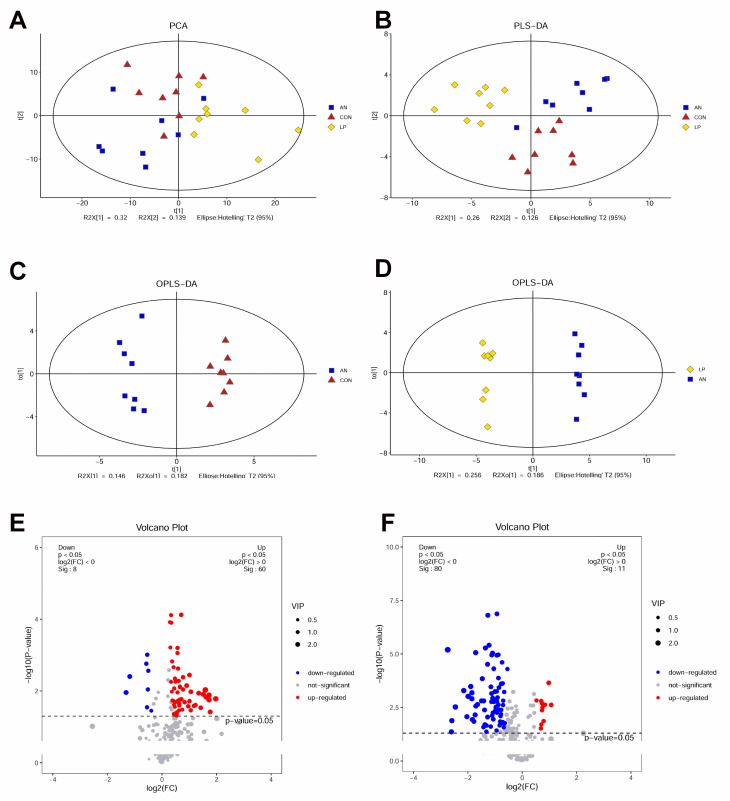
Effects of *Lactobacillus plantarum* supplementation on serum metabolome in broilers exposed to ammonia. (**A**) PCA, (**B**) PLS-DA, (**C**) OPLS-DA between AN and CON, (**D**) OPLS-DA between LP and AN, (**E**) Volcano plot between AN and CON, (**F**) Volcano plot between LP and AN. CON represents birds fed with basal diet, AN represents birds fed with basal diet and exposed into ammonia, LP represents birds fed with basal diet added *L. plantarum* and exposed into ammonia. *n* = 8.

The metabolomes of PCA and PLS-DA showed that all the samples were clustered by different treatments (Figure 8A,B). OPLS-DA indicated that samples from the three treatment groups were well separated from the other groups (Figure 8C,D). The volcano plot showed that AN treatment significantly downregulated 8 metabolites and upregulated 60 metabolites compared to the CON treatment group, whereas LP treatment significantly downregulated 60 metabolites and upregulated 11 metabolites compared to AN treatment (Figure 8E,F).

**Figure 9:**
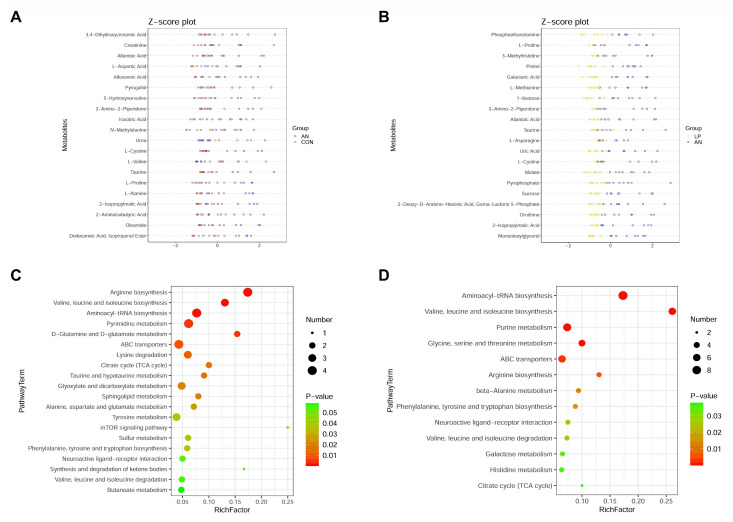
Effects of *Lactobacillus plantarum* supplementation on serum metabolome in broilers exposed to ammonia. (**A**) Z-score plot between AN and CON, (**B**) Z-score plot between LP and AN, (**C**) PathwayTerm analysis between AN and CON, (**D**) PathwayTerm analysis between LP and AN. CON represents birds fed with basal diet, AN represents birds fed with basal diet and exposed into ammonia, LP represents birds fed with basal diet added *L. plantarum* and exposed into ammonia. *n* = 8.

To separate the distinguished metabolites, the top 20 significantly changed metabolites according to the highest VIP values are shown in Figure 9. From the Z-score plot, amino acids (creatinine, cystine, valine, proline, methylalanine, and aspartic acid), oleamide, pyrogallol, allantoic acid, isocitric acid, aminoisobutyric acid, isopropylmalic acid, and taurine were significantly changed (*p* < 0.05) by AN treatment, whereas urea and valine were significantly decreased. LP administration significantly changed proline, asparagine, cystine, methylalanine, taurine, phosphoethanolamine, 3-methylhistidine, allantoic acid, uric acid, and ornithine levels.

Combined with the KEGG data, ammonia challenge significantly changed arginine, valine, leucine, isoleucine, biosynthesis, pyrimidine, alanine, glutamine, glutamate, taurine, hypotaurine, sphingolipid, tyrosine, butanoate, sulfur metabolism, lysine, valine, leucine, and isoleucine degradation compared with that of the CON birds (Figure 9C). LP supplementation significantly changed aminoacyl-tRNA, pantothenate and acetyl-coenzyme A (CoA), arginine, phenylalanine, tyrosine and tryptophan, valine, leucine, and isoleucine biosynthesis; purine, beta-alanine, galactose, histidine, alanine, aspartate and glutamate, glyoxylate and dicarboxylate, pyruvate, and thiamine metabolism; melanogenesis; and citrate cycle. 

No previous trial has been conducted to study the influence of LP treatment on serum metabolome changing in broilers challenged with ammonia. These metabolomic data are also consistent with the 16s sequencing data, in which ammonia changed arginine, valine, leucine and isoleucine, pyrimidine, alanine, glutamine and glutamate, taurine and hypotaurine, sphingolipid, tyrosine, butanoate, and sulfur metabolism, while LP supplementation significantly changed aminoacyl-tRNA, pantothenate and acetyl-coenzyme A, arginine, phenylalanine, tyrosine and tryptophan, valine, leucine and isoleucine biosynthesis, purine, beta-Alanine, galactose, histidine, alanine, aspartate and glutamate, glyoxylate and dicarboxylate, pyruvate and thiamine metabolism, melanogenesis, and citrate cycle compared to AN treatment.

## 4. Conclusions

Dietary *L. plantarum* supplementation decreased ammonia emissions by reducing serum urea nitrogen and ammonia, fecal urease, and ammonium nitrogen content. LP administration also improved the level of serum IgY, IgM, IL-10, T-AOC, and GSH-Px as well as cecal SCFAs, and decreased IL-1β, IL-6, and TNF-α. Moreover, LP supplementation increased the cecal microflora diversity of broilers, and significantly changed the tyrosine and tryptophan, valine, leucine, and isoleucine biosynthesis, alanine, aspartate, glutamate, pyruvate and thiamine metabolism, melanogenesis, and citrate cycle.

## Figures and Tables

**Table 1:** Composition and nutrient level of basal ration (air-dried level).

Item	Stage (d)	
	1–28	29–63
Composition (%)		
Corn	54	54
Soybean meal	23.2	15
Extruded soybean	5	3
corn lees	8.2	8
rice bran		9
corn-bran		2
Soybean oil	1.6	3.5
Limestone	1.4	1.5
fermented soybean meal	2.6	
Vitamin and mineral ^1^	4	4
Total	100.00	100.00
Nutrition level		
ME Kcal/kg diet ^2^	2915	3089
CP (%)	20.2	17.1
L-lysine HCl (%)	0.26	0.24
DL-methionine (%)	0.30	0.23
Ca (%)	0.86	0.74
Available *p* (%)	0.45	0.42

^1^ Vitamin and mineral premix supplied each kg of feeds with: Vitamin A, 1500 IU; Vitamin D3, 200 IU; Vitamin E, 10 IU; Vitamin K, 35 g; Vitamin B1, 1.5 mg; Vitamin B2, 3.5 mg; Vitamin B6, 3 mg; Vitamin B12, 10 μg; pantothenic acid, 10 mg; nicotinic acid, 30 mg; Biotin, 0.15 mg; Folic acid, 0.5 mg; Choline chloride, 1000 mg; Iron, 80 mg; Copper, 8 mg; Manganese, 60 mg; Zinc, 40 mg; Selnium, 0.15 mg; Iodine, 0.18 mg. ^2^ ME: metabolizable energy.

## Data Availability

Data presented are original and not inappropriately selected, manipulated, enhanced, or fabricated.
